# A Rare Case Report of Biloma After Cholecystectomy

**DOI:** 10.7759/cureus.5459

**Published:** 2019-08-22

**Authors:** Mohammed FaisalUddin, Roopam Bansal, Pulwasha M Iftikhar, Javidulla Khan, Azeem H Arastu

**Affiliations:** 1 Internal Medicine, Deccan College of Medical Sciences, Hyderabad, IND; 2 Internal Medicine, Sagar Gian Medical College, Patiala, IND; 3 Obstetrics and Gynecology, St. John's University, New York, USA

**Keywords:** biloma, biliary system, cholecystitis, cholelithiasis, cholecystectomy

## Abstract

Biloma is an encapsulated collection of bile outside or inside the biliary system within the abdominal cavity. It is a rare condition with an incidence of 0.3%-2%. The most common cause of spontaneous biloma is choledocholithiasis, and other causes include abdominal trauma and surgery, bile duct tumors, liver infarction, percutaneous catheter drainage, transhepatic cholangiogram and endoscopic retrograde cholangiopancreatography (ERCP) but the exact cause is yet to be discovered. We herein present a case report of biloma as a complication of laparoscopic cholecystectomy. A 58-year-old male presented to our hospital emergency room with complaints of fever, nausea, vomiting, and pain in the right upper quadrant after six weeks of laparoscopic cholecystectomy for cholecystitis. He was diagnosed with computed tomography (CT) scan quickly, and he has treated with pigtail catheter percutaneous drainage. On a follow-up visit, after four weeks, his abdominal pain had improved and white blood count was also reduced to baseline.

## Introduction

Biloma is an encapsulated collection of bile outside or inside the biliary system within the abdominal cavity [1-2]. It is a rare condition with an incidence of 0.3%-2%. Causes of biloma include traumatic biliary system injury, spontaneous rupture of the biliary tract and abdominal injury. After endoscopic cholecystectomy, the chance of biloma is 0.3%-0.6% [3-4].

The bile collection usually occurs after biliary surgery and the most common site is subhepatic space. More than 50% of biloma originates from the cystic duct, but after cholecystectomy, a rare subcapsular biloma can also be seen [1,5]. Biloma is uncommon without trauma, surgery, percutaneous transhepatic cholangiography (PTC) and endoscopic cholecystectomy, but if it occurs, there is high mortality and morbidity if not diagnosed early and treated promptly [4,6].

The clinical sign and symptoms usually occur in the first postoperative week of biliary surgery and the presentation varies from abdominal pain, jaundice, and fever to even peritonitis. An abdominal ultrasound is the first imagining modality to diagnose biloma but its equivocal computed tomography (CT) scan, magnetic resonance cholangiopancreatography (MRCP), and hepatobiliary iminodiacetic acid (HIDA) scan can also be done [2-3]. In terms of treatment, if the biloma is small and uncomplicated, it resolves spontaneously but in severe cases, endoscopic retrograde cholangiopancreatography (ERCP) and surgical hepatojejunostomy would be the treatment choice [2].

ERCP should be performed with biliary endoprosthesis and stent placement. The drainage of biloma may take 2-3 months. The broad-spectrum antibiotic therapy should be prescribed for 10-14 days from the first day of surgery [5-6]. We herein present a rare case of biloma in a 58-year-old male.

## Case presentation

A 58-year-old male presented to our hospital emergency room with complaints of fever, nausea, vomiting, and pain in the right upper quadrant. He also had a hospital visit three months ago due to abdominal pain, and he was diagnosed with acute cholecystitis based on the abdominal ultrasound which showed cholelithiasis without any evidence of gall bladder changes. He was treated conservatively and discharged home in a stable condition. Elective laparoscopic cholecystectomy was done six weeks after the initial visit. He denied any other medical and surgical condition.

**Figure 1:**
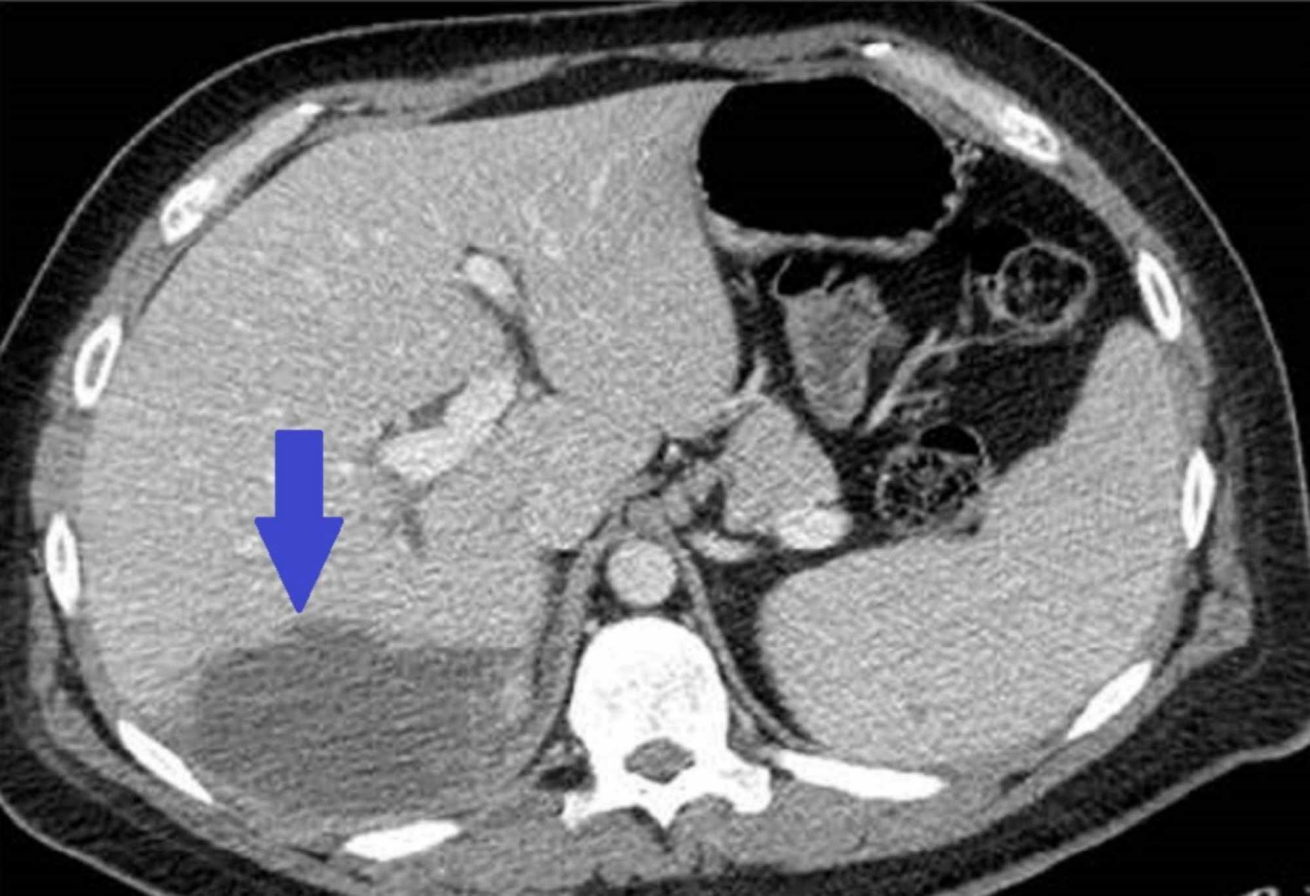
Abdominal computed tomography (CT) scan showing a rim enhancing subhepatic fluid collection

**Figure 2:**
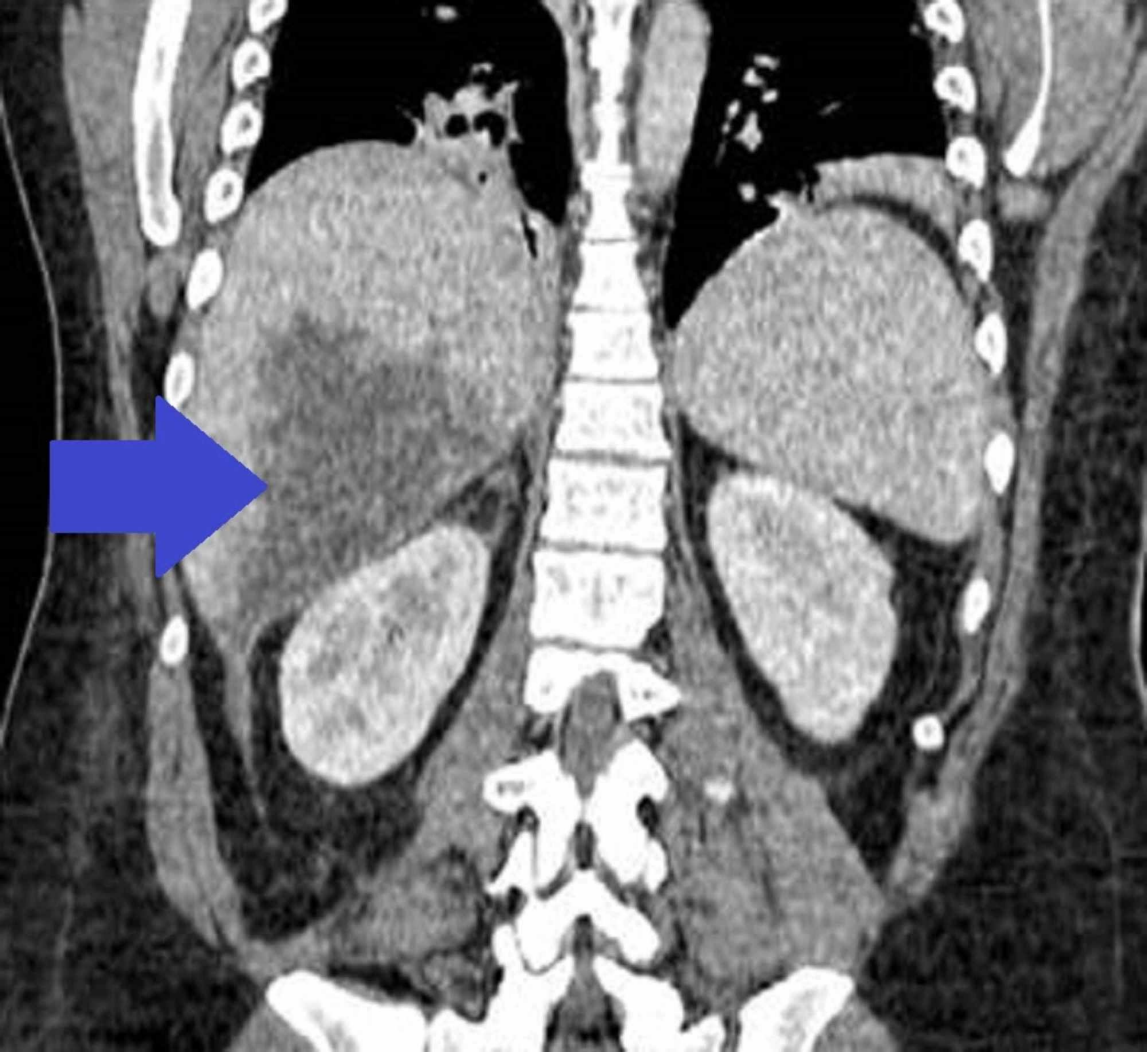
Computed tomography (CT) scan of the abdomen demonstrating a hypodense lesion in the right hepatic lobe

On clinical examination, he was in acute distress, his pulse was 116/bpm, he was febrile (101 F) and his respiratory rate was 25/min. On abdominal examination, he had mild epigastric tenderness without any signs of peritoneal irritation, and Murphy’s sign was negative. His initial blood work revealed a white blood cell count of 35,000/mm3, hemoglobin level of 12.9g/dl, and platelet count of 110,000/mm3. His liver function test (LFT) was elevated, alanine transaminase (ALT) of 155 IU/L, aspartate transaminase (AST) of 125 IU/L, alkaline phosphatase (ALP) of 310 IU/L, lactate dehydrogenase (LDH) 350 I/U, and normal bilirubin levels. However, his renal function test was normal, and his hepatitis serology was negative.Emergent ultrasound of the abdomen showed the well-circumscribed non-homogenous fluid collection in the right lobe of the liver without any changes in the gall bladder. CT scan of the abdomen was done to confirm the lesion and it showed 6.2×4.4×4.6 cm rim enhancing subhepatic fluid collection (Figures 1-2).

By history, clinical examination, and diagnostic tests, the diagnosis of biloma was confirmed. Percutaneous drainage was performed with a 7-Fr pigtail catheter by an interventional radiologist, and 800 ml fluid was drained on the first day. The patient was treated conservatively for five days and the drainage of fluid was monitored. On the sixth day, no drainage was noticed and the pigtail catheter was removed. His abdominal pain subsided, and white blood cell count decreased to 12,500/mm3. He was discharged home on oral antibiotics. After four weeks, a CT scan was repeated which showed the resolution of biloma with any other pathology. His clinical condition has improved gradually on a follow-up visit.

## Discussion

Loculated collection of the bile outside the biliary tract within the abdominal cavity is called biloma and the most common location is subhepatic space [7-8]. The most common cause of spontaneous biloma is choledocholithiasis, and other causes include abdominal trauma and surgery, bile duct tumors, liver infarction, percutaneous catheter drainage, transhepatic cholangiogram, and ERCP but the exact mechanism is yet to be discovered [9-10].

In 1971, the first case of a biloma was reported by Gould et al. and the patient had an abdominal trauma, resulting in extrahepatic bile leakage, and subsequently encapsulated biloma without any signs of peritonitis [11-12]. According to the study by Vazquez et al., bile collection is usually encapsulated when it occurs quickly in a short period and it can cause peritonitis, but if the leakage and collection occur slowly, there is only mild inflammation of biliary tract and peritoneum [13]. Similarly, in our case report, the patient developed encapsulated biloma as a consequence of cholelithiasis. Most reported cases of biloma are not spontaneous and usually accompanied by other diseases. During 1979- 1997, Fujiwara et al. reported 25 cases of spontaneous biloma and his study showed, there are various causes of biloma including obstructive jaundice, cholecystitis, cholangiocarcinoma, choledocholithiasis, liver abscess, tuberculosis and nephrotic syndrome [14]. In his study, 11 cases had biloma in the left hepatic lobe, 11 patients had right hepatic lobe involvement, and the remaining four cases had upper abdomen biloma. However, in our case report, the exact location of biloma (right hepatic lobe) was identified by CT scan. Nowadays, both intrahepatic and intraperitoneal collection of bile is called biloma [14].

The clinical presentation of biloma is variable and it could present with diffused or localized abdominal pain, fever, and jaundice. Rarely, it can cause ascites and peritonitis without fever. Elevated liver enzymes and leukocytosis may also be seen [1,5-6]. The exact size and site of biloma are directly affected by the cause of the bile tract injury, location, speed of bile leakage and rate of its absorption in peritoneum [9]. The clinical manifestations of biloma are variable and non-specific, and the diagnosis is based on clinical and radiological (ultrasound, MRCP, HIDA scan) findings. Sometimes the puncture of a cystic lesson or fluid aspiration analysis is required to confirm the diagnosis [1,5-6,11].

If biloma is small, no treatment is required and only observation is enough. The treatment depends on the severity of the disease. The surgery was the only main treatment option for biloma in the past but nowadays other options are available. Surgery can be done only for biloma with persistent leakage or for treating underlying disease [9]. Most postoperative bilomas are managed by percutaneous drainage with the placement of stent endoscopically or nasobiliary. If the drainage and conservative treatment with broad-spectrum antibiotic therapy fail, the advanced management with stent placement for prolonged drainage, micro-coil, and ethanol intrahepatic embolization would be the treatment options [7,8,10,14].

Biloma can be infected and cause serious and life-threatening complications such as peritonitis, biliopleural fistula which can lead to empyema, bilhemia (the fistula between veins and bile ducts inside liver, resulting in severely elevated bilirubinemia), and hemobilia (the arterial pseudoaneurysm rupture into the biliary system resulting in upper gastrointestinal hemorrhage) [8,14].

## Conclusions

Biloma is a rare medical condition. Early diagnosis and prompt treatment can prevent life-threatening complications. Physicians should be diligent enough to include biloma as a differential diagnosis when excluding other causes of right upper quadrant pain. CT-guided percutaneous drainage is an affordable treatment option for biloma with excellent results.
